# Medical liability litigation in Saudi Arabia

**DOI:** 10.4103/1658-354X.71133

**Published:** 2010

**Authors:** Abdulhamid Hassan Al-Saeed

**Affiliations:** *Professor in Anaesthesia & Critical Care Medicine College of Medicine, King Saud University, Chairman of Saudi Anaesthetic Association Member of the Legal Health Organization, Riyadh, Kingdom of Saudi Arabia*

**Keywords:** *Anesthesia*, *malpractice*, *medical litigation*

## Abstract

**Background:**

The author analyzed the anesthesia medical malpractice closed claims that were referred to the Legal Health Organization (LHO) in order to evaluate the magnitude and underlying factors of the problem in Saudi Arabia.

**Materials and Methods:**

Annual reports covering the period from 1420H–1429H (1999–2008) were statistically analyzed to give mean figures and percentages in each annual report, and then demonstrated all together to run the differential analysis together with the trend along the studied period.

**Results:**

Data analysis showed an escalating trend for the total number of claims over the study period being started with 440 cases on 1420H and ended with 1356 cases by the year 1429H. The annual percentage of the final verdicts of accusation to the total number of claims presented to all committees ranges between 45.5%–60.2% with a mean value of 49.9%. Distribution of final verdicts among different clinical specialities showed that obstetrics takes the lead with a mean percentage of 25.5% along the studied period (1420H–1429H), followed by the practice of general surgery with a mean percentage of 13.8%. The sector of health care service showed a significant variation in relation to the mean number of final verdicts with accusation along the studied period, being the highest in the Ministry of Health sector with a mean number of 216.8 claims, followed by the private sector with a mean number of 197.3 claims.

**Conclusion:**

Adherence to the standards of medical practice is by far to the best approach to avoid and reduce the incidence of litigation.

## INTRODUCTION

A “medical liability claim” is a claim or a cause of action alleging treatment or lack of treatment that departs from accepted standards of medical care which proximately results in the injury or death of a patient.[1]

Since the middle of the twentieth century, the medical profession has demonstrated an increase in the incidence and severity of medical liability lawsuits. Some feel that this rise in litigation is useful because learning from errors makes healthcare safer for the entire community and holds physicians accountable for their actions. However, opponents believe that litigation is unnecessary to maintain health standards, asserting that “nothing could be more damaging to the future of medical care than the suggestion that patients sue their doctors. Medicine, unlike other professions, it relies on a humans (physicians), rather than machines, to make complicated decisions that may have potentially severe and lifelong consequences. Adverse outcomes are often an inherent risk of medical care and do not necessarily reflect poor treatment.[1,2]

Health Care Services in Saudi Arabia have shown a great evolution over the past two decades in both governmental and private sectors. This development in health care was the result of the upgraded technology at the facilities as well as the training and improved experience of the medical practitioners. However, the increasing number of population together with the increased awareness about health matters resulted in an increasing trend of medical practice litigations. This was reflected by the number of complaints and claims against health care providers (whether generally as a facility or individually against physicians). Thus, to handle such an impact, it was found necessary to formulate and to set standards and regulations that determine the responsibilities of health care providers towards patients.[3]

The author is an active member of the Legal Health Organization (LHO) in Saudi Arabia (Riyadh region), and served for 10 years since the inception of LHO from 1999–2008 (1420H–1429H). The author has been actively involved in studying all the claims that reached the LHO (Riyadh region) and contributed to the final verdict (based on both professional standards and Islamic shariaah law) along with other committee members. In this report, the author has attempted to retrospectively analyze the various claims during a 10-year period and provide an insight on the working process of the LHO committee.

## MATERIALS AND METHODS

Data were collected from the official annual reports of Legal Health Organization; the data received were raw figures demonstrates a census of all claims presented to different committees covering the Kingdom of Saudi Arabia.[4]

The number of committees and additional subcommittees were 14 till 1428H (2007), and then upgraded to a total of 16 by 1429H (2008). Each subcommittee within the same region assigned to investigate a specific sector of health facility claims (Ministry of Health, Military, University, etc.,) and to end up with a final verdict of accusation, settlement or to file the whole case as being irrelevant from the plaintiff side.

Annual reports covering the period from 1420H–1429H (1999–2008) were statistically analyzed to give mean figures and percentages in each annual report, and then demonstrated all together to run the differential analysis together with the trend along the studied period.

Different aspects were included within the data of the annual reports; for example, the nationality of accused medical staff, gender, qualification, job title, also whether if the claim includes technicians and nursing or not, number of sessions conducted within each subcommittee to investigate the claims, but the author had concentrated on definite aspects that will identify the magnitude of litigations and its specification in relation to the sector of health service as well as the specialty of medical practice involved in the claim.

In the present study, statistical analyses were performed on the following data:

1. Annual number of claims presented to all committees
2. Ann ual number of final verdict with accusation
3. Distribution of final verdict with accusation among health sectors
4. Distribution of final verdict with accusation among different medical specialities.

## RESULTS

**Figure 1:**
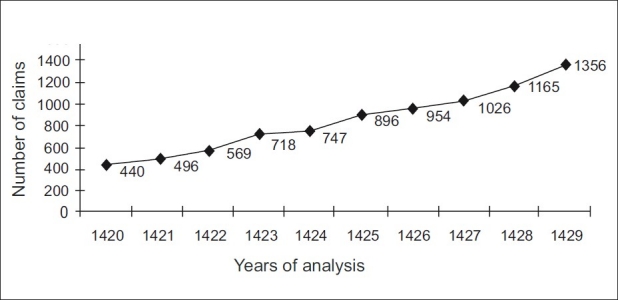
Trend analysis for the total number of claims presented to Legal Health Organization Committees all over the Kingdom (16 Committees)

Data analysis showed an escalating trend for the total number of claims over the study period being started with 440 cases on 1420H and ended with 1356 cases by the year 1429H [Figure 1].

The annual percentage of the final verdicts of accusation to the total number of claims presented to all committees ranges between 45.5%–60.2% with a mean value of 49.9% [Table 1].

**Figure 2:**
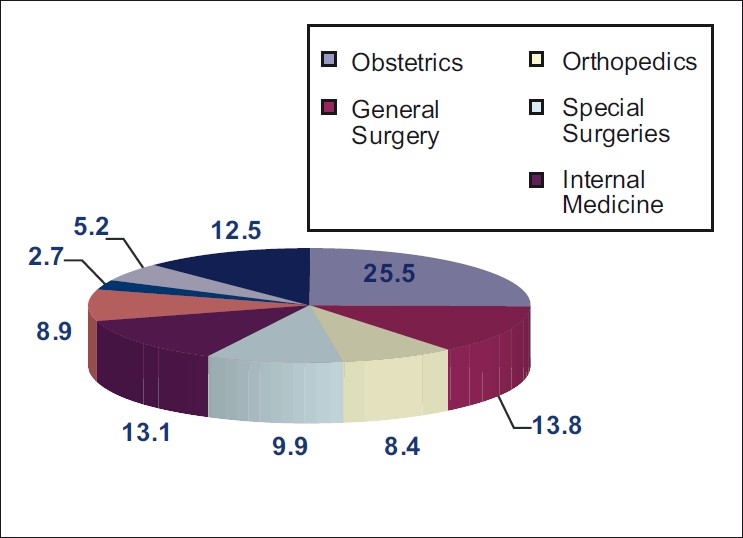
Mean percentage distribution of final verdict claims with accusation in different clinical specialties

Distribution of final verdicts among different clinical specialities showed that obstetrics takes the lead with a mean percentage of 25.5% along the studied period, followed by the practice of general surgery with a mean percentage of 13.8%. While among the special surgeries, orthopedic surgery found to have the highest percentage of claims among all other surgical specialities. Internal medicine and pediatrics follows the surgical specialities collectively with a mean percentage of final verdicts with accusation of 13.1% and 8.9%, respectively. The lowest incidence of accusation among all clinical practice specialities was the anesthesia speciality with a mean percentage of 2.7% along the studied period of 10 years [Figure 2].

**Table 1:** Percentage of the final verdict with accusation in relation to the total number of claims presented to all committees of Legal Health Organization

Data Years	Total number of presented claims	Final verdict of accusation	Percentage
1420	440	265	60.2
1421	496	255	51.4
1422	569	271	47.6
1423	718	393	54.7
1424	747	353	47.2
1425	896	428	47.7
1426	954	480	50.3
1427	1026	480	46.7
1428	1165	531	45.5
1429	1356	650	47.9

**Figure 3:**
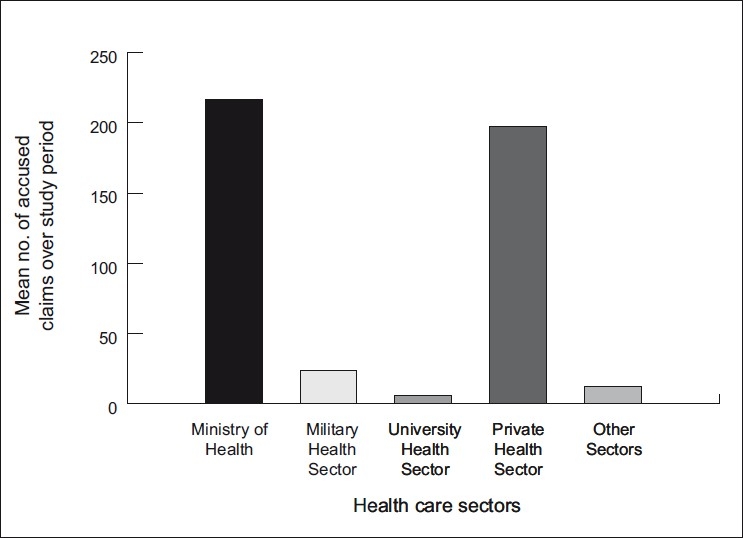
Mean number of final verdict claims with accusation in different health sectors

The sector of health care service showed a significant variation in relation to the mean number of final verdicts with accusation along the studied period, being the highest in the Ministry of Health sector with a mean number of 216.8 claims, followed by the private sector with a mean number of 197.3 claims. Military health care services showed a significantly lower number of accused claims in relation to the aforementioned sectors, being the mean number of accused claims 23.7 claims. While the lowest number was demonstrated among the university health care service hospital with mean number of accusation of 5.5 claims along the studied period [Figure 3].

## DISCUSSION

Litigation does have its merits as a form of dispute resolution. Perhaps the single best quality of litigation is that it seeks to identify an individual or group whose negligent actions resulted in harm to the patient. It holds doctors accountable for their decisions. Because no reasonable doctor wants to be found guilty of harming his patients, litigation or rather the threat of litigation encourages doctors to practice carefully. Thus, litigation, at least in theory, protects patients.[1,5]

In the Kingdom of Saudi Arabia, the process of medical litigations starts once a patient or a member of his/her relatives complains of a medical malpractice from their point of view that ends with a morbidity or mortality. The complaint is directed either to the Ministry of health or the City Government according to the medical facility involved in the complaint. A process of investigation and interrogation follows within the medical facility with the medical staff either sharing the responsibility or attending the event. LHO then assigns to follow with a process of thorough review of all documents and medical filling together with interviewing both sides of the claim “the plaintiff and defendant(s)”, in order to reach a final verdict of accusation or clearance from the claim according to the “Regulations of Medical Practice,” which is based on professional aspects and governed by the Islamic Shariaah law. Professional liability as an entity covers 3 different aspects:

1. The Civil liability: This is the responsibility of a physician towards the patient when harm is being inflicted as a result of direct action against medical rules from the physician or proven negligence.
2. The Punitive liability: This deals with physicians who violate the rules and regulations of medical practice even with no subsequent harm resulted to the patient.
3. The Disciplinary liability: Wherein a physician failed to meet with professional standards, requirements, and ethics.[3]

Finally, the claim if reaching a final verdict with accusation may end up in warning and/or financial compensation, according to the Islamic Shariaah law, prohibiting medical practice or withdrawal of medical license.[3]

The committees of legal health organization are composed primarily of a judge with Islamic shariaah background to reach the verdict after completion of the medical investigation of the claim that is the responsibility of the other 3–4 members with the medical background referring to different sectors of health service mainly from ministry of health and university staff members.[4]

One corner stone of the Islamic shariaah law states that it compensates for any disability, morbidity, or mortality resulting from proved negligence or malpractice of medical intervention, while providing that the medical staff indulged being qualified, licensed, and experienced to perform such intervention. Here, the point that matters is not just the occurrence of a complication, but how proactively and professionally it was managed.

Data analysis revealed a significant increase in the number of claims presented to different committees of LHO, while consistently keeping the percentage of final verdict with accusation of negligence or malpractice within the same range of around 49.9% of the total number of cases investigated being either cleared or filed for irrelevance.

Importantly, it is widely accepted that the rise in litigation is not due to an increasing incidence of clinical negligence, but rather “the increasing tendency of patients to seek legal redress and the rising costs of such legal settlements”. So this could be proved by the percentage aforementioned regarding accused verdicts. However, the fact of increased litigations stems from people who became more aware of standard medical care and demanding for it as well.[6]

On the other hand and with regards to the increasing number of claims, that results in a lengthy process of pending cases being investigated to reach a verdict with the consequence of time and cost expenditure.

On the administrative side and as a reflection to increased number of claims, the Saudi council for health specialities set a rule of not providing the medical license to any doctor unless submit his authentic certificate of medical insurance against malpractice, thus to ensure that settlement compensation could be fulfilled once the verdict declared by the LHO.

Another aspect of data analysis was targeted towards the incidence of clinical specialty for claiming, which showed that generally surgical specialities are significantly higher than medical specialities; this could be explained by the extent of procedural intervention in surgical field rather than the medical field and lower incidence for complications.

Anesthesia has been classified as a high-risk specialty. This classification is based on the fact that the state of induced hypnosis may result in airway obstruction, pulmonary aspiration or trauma. Most anesthetic drugs have undesirable adverse effects on both cardiovascular and respiratory systems. Further, an anesthetized patient is totally dependent on the anesthesiologist and equipment for maintenance of his vital functions. Thus, being an anesthesiologist it was possible to further analyze and concentrate on the scope of anesthesia-related claims and its relationship to the total number of claims.

This contributes the lowest percentage among all clinical specialities. Different articles and meta-analysis studies worldwide have navigated the scope of anesthesia-related malpractice and conclude strongly that cardiorespiratory arrest and cerebral damage resulting from hypoxemia is the leading cause that ends in mortality or drastic morbidity. Oxygen supply to the patient being of the highest concern rather than any defect in alveolar gas exchange or oxygen delivery to the tissues, meaning equipment failure or matters dealing with a compromised upper airway with the inability to adequately ventilate a hypnotized, sedated and/or paralyzed patient.[3]

Neuroaxial deficits resulted after regional anesthesia techniques was the second common cause, but with a wide range of consequences that being simple as transient neurapraxia up to permanent loss of function resulting from peripheral nerve damage or spinal cord injury. Also, lawsuits against intra-operative awareness are not uncommon with its psychological feedback on patients in the postoperative period.[7,8]

Following international standards could restrict the magnitude of medical errors which had been classified by the Agency for Health Research and Quality as diagnostic error, equipment error, misinterpretation of medical orders or data and finally mismanagement with resultant morbidity as postoperative infections or mismatched blood transfusion, all of which could be easily applied to the field of anesthetic practice.[9]

Thus, it will not be incorrect to mention that the medical facility plays an important role in the increased incidence for litigations. Data analysis revealed that the ministry of health and private sectors both together contributes more than 90% of the total number of claims that referred to the LHO. The Ministry of Health (MOH) remote hospitals or small clinics covers most of the small cities and that most of those facilities are run by under-trained and understaffed physicians together with inadequate equipment and supplies, a fact which renders such facilities more prone for malpractice and litigations. The private secto is mostly well equipped and staffed with qualified and experienced practitioners, and the patients going to this expensive sector consider their culture and social class more demanding to quality of health care service.

In general, malpractice litigation has three social goals: to deter unsafe practices, to compensate individuals injured through negligence, and to exact corrective justice. Medical malpractice lawsuits are intended to deter physicians from providing substandard care by reminding them that if they wish to avoid the emotional and financial costs of litigation, they must take care to practice safe and effective medicine. With respect to compensation, it seems reasonable that the party at fault for an injury should bear the associated costs, including lost earnings, medical bills, and costs associated with “pain and suffering.”[1]

“Policies and Procedures”, “Rules and Regulations”, “Standards of Medical Practice” all grouped falls into the same concern that is quality assured medical service that ensures patient safety.[3] But there are some fundamentals for dealing with claims during investigation, which stems to ensure justice between both sides of the case. A few of them are being described below:

1. Dealing with knowledge: A physician was held legally responsible for what he said he was able to do.
2. Nature of the skill: Ordinary, not extraordinary, skill was all that was required by law. That profession of physician and surgeon must be held to employ a reasonable amount of care and skill. For anything short of that degree of skill in his practice, the law will hold him responsible for any injury which may result from its absence. While he is not required to possess the highest order of qualification, to which some others attain, still he must possess and exercise that degree of skill which is ordinarily possessed by members of the profession.
3. Nature of care: Physicians were expected to use reasonable and ordinary care in their application of their knowledge and skill. It was not assumed that in all events, patient care would occur safely and without injury.
4. Dealing with mistakes or errors of judgment: The physician who exercised his best judgment was not responsible for errors of judgment or mistakes where there were reasonable grounds for diagnostic doubts and differences of opinion about treatment.
5. Opinions about cure: Legally, physicians were not required to guarantee or ensure a cure. However, the law would not support a malpractice defence by a physician if an absolute cure had been promised.[10]

In conclusion, this analysis ends up with definite facts, that on the governmental level and as a result of the significant high incidence of claims against MOH sector of health services, resources had to be directed more towards this sector to improve the quality of medical service provided. While on the level of the process of litigation against MOH and private sector claims and within the LHO, here number of committees investigating these sectors had to be increased to match with the increased number of litigations and thus to save the waste of time and money expenditure taken for investigating cases for years in some incidents. On a professional level, my only advice is that when you are certain that the consequences of an error are disastrous so it is of logic to be too careful.
